# Supplementary material for: Slope stability assessment in the seismically and landslide-prone road segment of Gerese to Belta, Rift Valley, Ethiopia
Source: PLoS One. 2024 Feb 13;19(2):e0296807. doi: 10.1371/journal.pone.0296807 (PMC10863894; doi:10.1371/journal.pone.0296807)
Supplement: Attachment — Submitted filename: Response to Reviewers.docx [file pone.0296807.s001.docx]

Dear Editor-in-Chief, PLOS ONE

I would like to thank you for allowing me to revise my manuscript " Slope Stability Assessment in the Seismically and Landslide-Prone Road Segment of Gerese to Belta, Rift Valley, Ethiopia" PONE-D-23-31776. I am grateful for the insightful feedback provided by the reviewers and the opportunity to address their comments and suggestions. I would like to express my sincere appreciation to the reviewers for their valuable input, which has significantly enhanced the quality and clarity of my work. Their constructive comments have enabled me to refine my research and strengthen the overall contribution of the article.

I have carefully considered the reviewer's suggestions and made the necessary revisions to address their concerns.

I am herewith providing a point-by-point response to each reviewer's comment.

Academic Editor Comments

In your Methods section, please provide additional information regarding the permits you obtained for the work. Please ensure you have included the full name of the authority that approved the field site access and, if no permits were required, a brief statement explaining why.

Author’s response: The current research was carried out during the ongoing road construction for public use, eliminating the necessity to obtain permission for collecting soil and rock samples in that area.

In your Data Availability statement, you have not specified where the minimal data set underlying the results described in your manuscript can be found.

Author’s response: All field and laboratory data utilized in this study have been incorporated within the paper, and a Data Availability Statement has been included in the revised version of the paper.

We note that Figure(s) 3, 7, 8, 9, 10, 11, 12, 13b, 14b, 15b and 16b in your submission contain copyrighted images.

Author’s response: The aforementioned figures were omitted in the revised paper.

We note that Figure 1 and 2 in your submission contain [map/satellite] images which may be copyrighted.

Author’s response: The authors created Figure 1. And citation mentioned for figure 2.

Reviewer #1:

1. The Abstract should be concise to highlight the main subject matter and aspect of the paper with brief findings.

Author’s response: As per your instructions, the abstract has been rewritten and updated in the revised paper.

2. In the literature review portion, several important and recent relevant contributions are missing.

Author’s response: Updated the revised paper with additional (references 30 to 37), pertinent, and recent references.

3. In the Materials and methods portion, it is lack of results of lab test and field survey.

Author’s response: The laboratory test outcomes and field survey findings are displayed in subsections 2.4.2 and the annexure within the revised paper.

4. In the slope stability analysis portion, the content should be reorganized to support the research.

Author’s response: In the updated paper, elucidate the research findings within subsections 3.2.3 and 3.2.4.

5. In the Remedial measure portion, there are no effective solutions for this problem.

Author’s response: In the updated paper, elucidate the distinct remedial measures within subsections 3.4.1, 3.4.2, 3.4.3, 3.4.4, and 3.4.5.

6. The conclusion should be concise with focus on the primary research findings.

Author’s response: Re written concisely in the revised paper.

7. The manuscript should have written in a academic paper style.

Author’s response: The manuscript underwent revisions in response to the reviewer's comments.

Reviewer #2: The paper performs studies on slope stability assessment in the seismically and landslide-prone road segment of gerese to belta, rift valley, Ethiopia, in which tests and numerical analysis were done. The contents are interesting. The paper can be accepted for publication only if the following comments are addressed.

(1) When performing the shear tests, how do you predict the parameters are applicable to the slope analysis?

Author’s response: During shear tests, parameters such as shear strength, soil properties, and results from laboratory tests are examined to understand their relevance to slope analysis.

(2) In 2.4.2.4 Limit Equilibrium Method, how do you obtain the effective strength parameters?

Author’s response: In the Limit Equilibrium Method used for slope stability analysis, effective strength parameters are typically obtained through laboratory testing and field investigations. The effective strength parameters crucial for this method include the cohesion (c) and the friction angle (φ) of the soil or rock mass

(3) In 3.1, How do you consider the seismic effects?

Author’s response: In the Ethiopian Rift Valley, considering seismic effects in slope stability analysis involves assessing the region's seismic hazard by studying historical seismic data and local ground motion parameters. Understanding the dynamic properties of soils and rocks, along with the geological conditions of slopes, is crucial.

(4) Do you consider the influence of rain infiltration?

Author’s response: Yes considered.

Once again, I would like to express my gratitude for the reviewers' insightful comments and the opportunity to revise and resubmit my work. I sincerely hope you and the editorial board will find the revised manuscript satisfactory for publication in the Egyptian Journal of Remote Sensing and Space Sciences. Thank you for considering this revised submission. I look forward to hearing from you regarding the outcome of the review process.

Thank you for your time and consideration.
